# P-1060. *In vitro* Activity of Gepotidacin Tested Against Molecularly Characterized *Escherichia coli* Isolates Responsible for Urinary Tract Infections in the US (2019-2022)

**DOI:** 10.1093/ofid/ofae631.1249

**Published:** 2025-01-29

**Authors:** Rodrigo E Mendes, Danielle Beekman, Abigail Scullin, Maura Karr, Renuka Kapoor, Didem Torumkuney, S J Ryan Arends

**Affiliations:** JMI Laboratories, North Liberty, Iowa; Element Materials Technology/Jones Microbiology Institute, North Liberty, Iowa; Element Materials Technology/Jones Microbiology Institute, North Liberty, Iowa; Element Materials Technology/Jones Microbiology Institute, North Liberty, Iowa; GSK, Atlanta, Georgia; GSK, Atlanta, Georgia; JMI Laboratories / Element, North Liberty, Iowa

## Abstract

**Background:**

Gepotidacin (GEP) is a novel, bactericidal, first-in-class triazaacenaphthylene antibacterial that inhibits bacterial DNA replication by a unique mechanism of action, distinct binding site and provides well-balanced inhibition (for most uncomplicated urinary tract infections [uUTI] uropathogens and *Neisseria gonorrhoeae*) of two different type II topoisomerase enzymes GEP completed two phase 3 trials for treatment of uUTI This study reports the activity of GEP and other oral antibacterials against *E. coli* (EC), including molecularly characterized isolates carrying ESBL and carbapenemase genes collected from UTI patients in the US.

**Figure:**
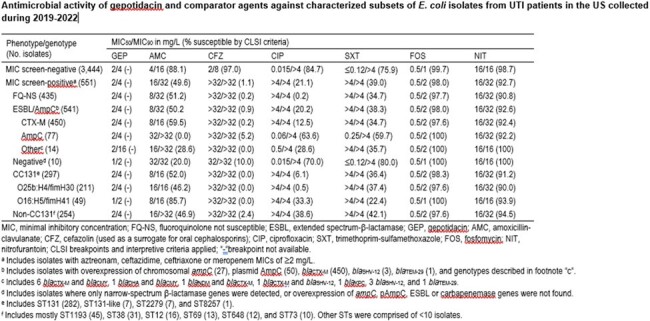

**Methods:**

3,995 EC collected from 52 US sites as part of the gepotidacin uropathogen global surveillance study were included (2019-2022). CLSI methods were used for susceptibility (S) testing and MIC interpretations. Isolates with aztreonam, ceftazidime, ceftriaxone or meropenem MIC of ≥2 mg/L were subjected to genome sequencing, and screening of β-lactamase genes and epidemiology typing (MLST, O:H, and *fimH*).

**Results:**

A total of 86.2% (3,444/3,995) of EC did not meet the MIC criteria for molecular characterization (Table) and had GEP MIC_50_ and MIC_90_ values of 2 mg/L and 4 mg/L, respectively. Cefazolin (97.0%S), fosfomycin (FOS) (99.7%S), and nitrofurantoin (NIT) (98.7%S) had activity among oral comparator agents tested against this group. A total of 13.8% (551/3,995) of EC were screened for β-lactamase genes, and this group had GEP MIC_50_ and MIC_90_ values of 2 mg/L and 4 mg/L respectively. Other oral agents had limited activity, except for FOS (98.0%S) and NIT (92.7%S). In general, GEP retained activity with MIC_50_ and MIC_90_ values of 2 mg/L and 4 mg/L, respectively, against isolates carrying ESBL, AmpC and carbapenemase genes, and against clonal complex (CC) 131 and the resistant subset O25b:H4.

**Conclusion:**

GEP showed generally consistent activity against UTI-causing EC in the US, including against isolates carrying ESBL, AmpC and/or carbapenemase genes. In addition, GEP had activity against the CC131 high-risk EC clone. These data support further clinical development of GEP as a potential treatment option for uUTI caused by EC including when other oral treatment options are limited due to drug resistance.

**Disclosures:**

**Rodrigo E. Mendes, PhD**, GSK: Grant/Research Support **Renuka Kapoor, PhD**, GSK: Employee|GSK: Stocks/Bonds (Public Company) **Didem Torumkuney, PhD**, GSK: Employee|GSK: Stocks/Bonds (Public Company)

